# Bond Performance of Sand Coated UHM CFRP Tendons in High Performance Concrete

**DOI:** 10.3390/polym9020078

**Published:** 2017-02-22

**Authors:** Tobias Dominik Lämmlein, Francesco Messina, Michele Griffa, Giovanni Pietro Terrasi, Pietro Lura

**Affiliations:** 1 Mechanical Systems Engineering Laboratory, Empa, Swiss Federal Laboratories for Materials Science and Technology, Überlandstrasse 129, 8600 Dübendorf, Switzerland; Giovanni.Terrasi@empa.ch; 2 Institute for Building Materials (IfB), ETH Zurich, 8092 Zurich, Switzerland; Pietro.Lura@empa.ch; 3 Departement of Engineering, Pathenope University of Naples, 80143 Naples, Italy; francesco.messina@uniparthenope.it; 4 Concrete and Construction Chemistry Laboratory, Empa, Swiss Federal Laboratories for Materials Science and Technology, Überlandstrasse 129, 8600 Dübendorf, Switzerland; Michele.Griffa@empa.ch; 5 Center for X-ray Analytics, Empa, Swiss Federal Laboratories for Materials Science and Technology, Überlandstrasse 129, 8600 Dübendorf, Switzerland; 6 Institute for Infrastructure & Environment, School of Engineering, University of Edinburgh, EH9 3FB Edinburgh, UK

**Keywords:** CFRP, UHM carbon fibre, UTS carbon fibre, prestressing tendon, sand coated, bond, HPC, pull-out test, finite element modelling (FEM), X-ray tomography

## Abstract

The bond behaviour of novel, sand-coated ultra-high modulus (UHM) carbon fibre reinforced polymers (CFRP) tendons to high performance concrete (HPC) was studied by a combined numerical and experimental approach. A series of pull-out tests revealed that the failure type can vary between sudden and continuous pull-out depending on the chosen sand coating grain size. Measuring the same shear stress vs. tendon draw-in (τ-δ) curves in the same test set-up, for sand coated CFRP tendons with a longitudinal stiffness of 137 and 509 GPa, respectively, indicated that the absolute bond strength in both cases was not influenced by the tendon’s stiffness. However, the τ-δ curves significantly differed in terms of the draw-in rate, showing higher draw-in rate for the UHM CFRP tendon. With the aid of X-ray computed tomography (CT), scanning electron microscopy (SEM) and visual analysis methods, the bond failure interface was located between the CFRP tendon and the surrounding sand-epoxy layer. For further investigation, a simplified finite element analysis (FEA) of the tendon pull-out was performed using a cohesive surface interaction model and the software Abaqus 6.14. A parametric study, varying the tendon-related material properties, revealed the tendon’s longitudinal stiffness to be the only contributor to the difference in the τ-δ curves found in the experiments, thus to the shear stress transfer behaviour between the CFRP tendon and the concrete. In conclusion, the excellent bond of the sand-coated UHM CFRP tendons to HPC as well as the deeper insight in the bond failure mechanism encourages the application of UHM CFRP tendons for prestressing applications.

## 1. Introduction

Slender CFRP prestressed concrete elements have beneficial properties compared to steel prestressed structures. These elements are much lighter, mostly due to the fact that CFRP does not corrode and the concrete cover can be reduced at a minimum dictated only by statics and ease of fabrication [1].

Current applications of slender, CFRP-prestressed elements are mainly used for building façades or as free-standing lighting poles [2]. The concrete used in these elements [1,2,3] is usually a high performance concrete (HPC). This choice of material accounts for high loads and facilitates the best possible utilization of the prestressing technique due to its high elastic modulus and low creep coefficient. In addition, the use of HPC also minimizes loss of prestress due to creep, which would lead to considerable loss of prestress in the UHM tendons with time and temperature. In these applications, the elements need to be anchored to a primary structure. The mounting of these elements can only be applied in their regions where the prestress is fully developed. Thus, the prestress transfer length, starting at the end of each element, should be kept as short as possible to reduce the dead weight and the non-usable area of the elements.

In CFRP, a wide choice of carbon fibres can be applied. These fibres vary largely in terms of longitudinal stiffness (230–827 GPa) and tensile strength (1400–7060 MPa) [4]. With a higher stiffness of the tendon, more loads can be transferred at a lower strain. Thus, it is proposed that the transfer length of prestressed elements will also be affected and may be shortened by using stiffer tendons. This would allow a further reduction of material and thus would lead to lighter and more functional designs.

Several studies investigated the stress transfer behaviour between tendons and the surrounding concrete [5,6,7,8,9,10], which is most important when the dead weight of the elements should be reduced. It was observed that the bond of prestressed tendons is one of the key elements during the design of load introduction and anchorage areas [11].

The bond behaviour of prestressing steel tendons and strands (pretensioning technique) has been studied intensively since the early 1950s (see, e.g., [11,12,13]). However, there is still little knowledge of the bond behaviour of CFRP tendons in high strength concrete and high performance self-compacting concrete (HPSCC). In contrast to standardized steel reinforcing tendons, the material and surface variants of FRPs are much more manifold and difficult to standardize [14]. Consequently, the possible influences on bond of FRP tendons are various. The effects of the tendon’s diameter [15], the bond length [16], the concrete strength [15,17] and the surface characteristics [18,19] on the bond are of major importance [20,21]. Surface features like embedded sand grains were found to be the main contributors to the bond between FRP reinforcements and concrete [22]. The bond of a sand-coated CFRP profile is principally based on adhesion and mechanical interlock between the rough surface of the tendon and its concrete cover. The force transfer of sand-coated FRP bars in concrete [23] showed a development length of only 16 tendon-Ø, significantly shorter than steel bars (~80–120 tendon-Ø). Thus, a sanded tendon’s surface is expected to be a good coating option which should assure a durable prestress as well as a short transfer length [24]. Indeed, sand-coated CFRP tendons proved to be effective at least up to 18 years in outdoor four-point bending creep tests [25]. Another benefit of the sand-coated surface is the application to a clean and intact CFRP tendon’s surface. In contrast to some alternatives, e.g., machined grooves on the CFRP surface, the tensile strength of the tendon is not lowered by the sand coating. Thus, this technique allows the best possible use of the beneficial CFRP properties.

One study exists dealing with the influence of tendon stiffness on bond between two types of glass fibre reinforced polymer (GFRP) tendons and concrete [26]. However, the GFRP tendons used in that study had longitudinal stiffness values of either 48 or 64 GPa, which is much lower than generally-used CFRP counterparts. The known studies on bond or prestress transfer of CFRP tendons had a longitudinal stiffness in the range of 115–167 GPa [16,17,18,19,24], substantially lower than steel (about 210 GPa).

None of these studies differentiate in detail the possible influences on the bond between diverse CFRP materials, in terms of their longitudinal tendon’s stiffness. The UHM-CFRP tendons used in this study reach longitudinal stiffness values of 509 GPa, which is 3–4.5 times stiffer than the mentioned currently-used CFRP tendons and almost 2.5 times stiffer than steel. It is anticipated that this increase in tendon stiffness will heavily influence the stress transfer behaviour between concrete and tendon, thus it needs to be characterized in detail.

The aim of this work is to investigate experimentally the effect of very high tendon stiffness on the bond with reference HPC. Moreover, the τ-δ-curves as well as the failure surfaces were studied and the information implemented within a FEM model in Abaqus 6.14 (Dassault Systemes Simulia Corp., Providence, RI, USA). This new knowledge about bond will form the basis for the continuation of this research, which focuses on the bending behaviour of UHM-CFRP prestressed structural elements.

## 2. Materials and Methods 

This work consists of three experimental and one numerical analysis sections. The used materials, their characterization as well as the applied test procedures are outlined here.

### 2.1. Raw Materials and Composites

An industry based high performance self-compacting concrete (HPSCC) was chosen for the first part of this work to investigate the bond strength of different sand coating options. This concrete mixture (*w*/*b* = 0.37), which was slightly further developed from [24], mainly consists of ordinary Portland cement CEM I 52.5 R, fly ash, silica fume, sand 0/4, gravel 4/8. PP-fibres, SIKA ViscoCrete20HE superplasticizer (SP) and stabilizer were also used. The basic mechanical properties in compression were tested according to EN12390 [27]. The corresponding tensile properties were measured on specimens slightly adapted and customized in geometry from SIA 2052 [28]. The properties are summarized under the name C_ind_ in Table 1. Furthermore the influence of the longitudinal stiffness of the CFRP tendon on the bond was studied with the same concrete as well as with a slightly adapted mixture. The adapted mixture, named C1 (*w*/*b* = 0.35), had only minor differences to the previously employed C_ind_. C1 used as well ordinary Portland cement CEM I 52.5 R and comparable amounts of sand 0/4 and gravel 4/8. In contrast to C_ind_, C1 did not include any stabilizer and PP-fibres. The basic mechanical properties of C1 were characterized as described above for C_ind_ (see Table 1) and were found to be very close to those of C_ind_.

In this study, a total of three different CFRP tendons were investigated. The classic production process for unidirectional (UD) CFRP tendons is pultrusion. The carbon fibres are impregnated with epoxy resin and afterwards pulled through a forming and curing die to obtain a circular cross section. An alternative way to produce CFRP tendons is a tape-laying method. To form the circular shape, a thin shrinking foil is wrapped around the laid down and slightly tensioned CFRP prepregs. Curing of this prepreg tendon takes place afterwards by applying heat. The advantage of the latter method is flexibility in terms of batch sizes and changes in materials. The tendons used in this study were produced, as mentioned later, by both of these methods.

The first type is a reference tendon used in industry [2] and was produced by pultrusion. This tendon consists of PAN based carbon fibres from type TENAX^TM^ UTS 5631 (Toho Tenax, Chiyoda, Tokyo, Japan) and a Bakelite Ruetapox VE 4434 (Hexion GmbH, Iserlohn-Letmathe, Germany) epoxy resin. This ultra-high tenacity strength (UTS) CFRP tendon had a diameter of 5.5 mm, a fibre volume content (FVC) of 64.5% and a relatively low longitudinal stiffness of only 137 GPa.

To assess the bond strength between UHM-CFRP tendons and concrete, two further tendon types were selected. Ultra-high modulus (UHM) PITCH based Mitsubishi DIALED^TM^ carbon fibres (Mitsubishi Rayon Co. Ltd., Chiyoda, Tokyo, Japan), K63A12 and K13916, were chosen as the two different fibre materials for these tendons. Both UHM-fibres were combined with a Huntsman XB3515/AD5021 hot-melt epoxy system to a prepreg. The two tendons were produced in the tape-laying method by Carbolink AG in Fehraltdorf, Switzerland. Both UHM-CFRP tendons had a diameter of 5.3 mm but a slightly different longitudinal stiffness of 464 GPa (K63A12) and 509 GPa (K13916), respectively. A summary of the characterized mechanical properties of all three tendons can be found in Table 2.

The CFRP tendons were coated by three different types of quartz sand particles to enhance the bond between the tendons and the concrete. The first two sand types, “fine” and “old-coarse”, were reference coatings used in industry [2] and during earlier work at Empa [25]. In both cases, the sand particles were applied to the tendon’s surface by spray coating in an industrial process directly after pultrusion. The third sand coating, named “new-coarse”, was chosen to further improve the bond strength. This sand coating was applied separately by a sprinkling technique following the tendon production. In this case, a thin layer of Huntsman 5052 epoxy resin was used to glue the sand particles effectively to the tendons surface.

**Scheme 1:**
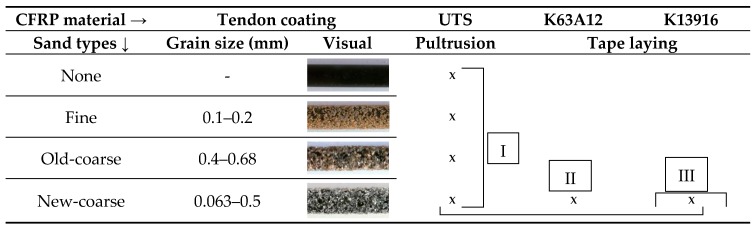
Sand coating characteristics and their application in this work.

All sand particles were characterized in size and visualized after their application, see Scheme 1. Furthermore, an overview of the tendon-sand-concrete combinations utilized in the different experimental sections (I–III) of this work can be found in the right part of Scheme 1. Here, (I) represents the determination of sand coating influence; (II) stands for the CFRP material’s influences on bond; and (III) investigates the bond failure by the aid of X-ray CT and measures the tendon draw-in/-out for UHM-CFRP tendons in a pull-out experiment.

### 2.2. Test Specimens

All pull-out tests were performed on 40 × 40 × 160 mm^3^ prisms. This specimen geometry is different from the usual cube type found in the literature [21,30,31]. However, in real applications of slender CFRP-prestressed structures, the typical element thickness lies in the range 40–60 mm. Hence, the chosen cross-section in this study should represent a conservative geometry related to the end-goal application.

**Figure 1:**
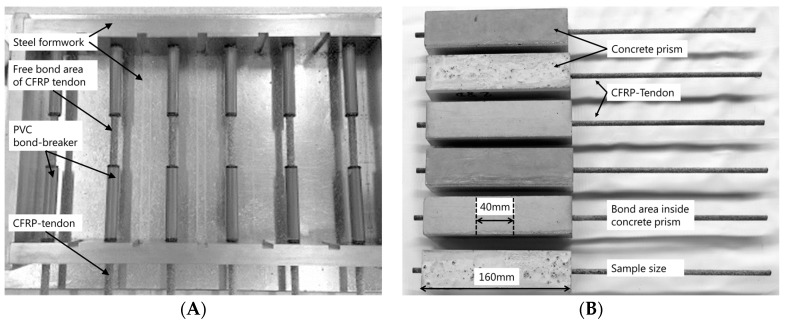
Specimen details: (**A**) CFRP-tendon and bond-breaker arrangement before casting; and (**B**) demoulded samples before testing.

The bond length was set to be 40 mm and kept equal in all specimens during this study. To enhance the measurement of high bond stresses, as proposed by Losberg [32], the bond length was defined to be in the middle of the longitudinal direction of the specimen. The unbonded area of 60 mm at each side of the bond area was created by the use of PVC-tubes. These PVC-tubes were sealed by a rubber O-ring to prevent direct concrete-to-tendon contact. The placement of the CFRP-tendons inside a steel mould is shown below in Figure 1A. After casting, the specimens were demoulded and stored in 90% RH and 20 °C until testing. An example of the finished pull-out samples can be seen in Figure 1B.

### 2.3. Experimental Pull-Out Setup

**Figure 2:**
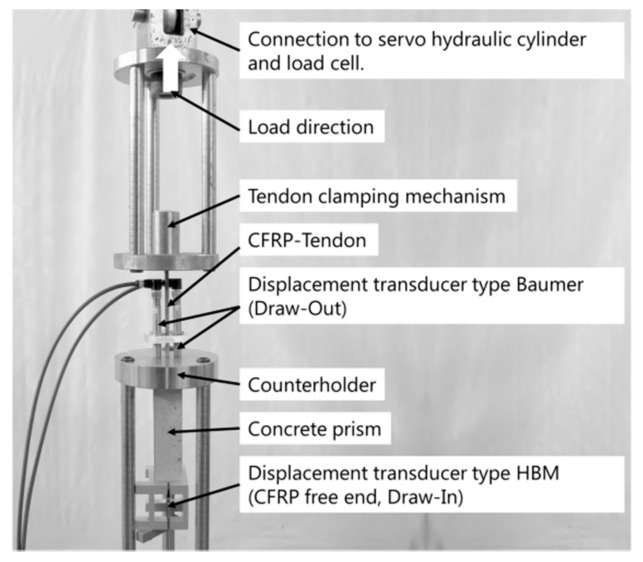
Pull-Out test configuration: Overview of mounted sample in experimental set-up.

To compare the influence on the maximum bond strength of different parameters, such as sand-coating and tendon stiffness, the proposed experimental set-up in EN10080 Appendix D was chosen and slightly adapted in geometry after considerations in [14,32]. Due to the long un-bonded distance of 60 mm between the counter holder and the 40 mm long bond area in the middle of the concrete prism, it is anticipated that the influence of concrete confinement due to friction at the counter holder is minimal. This was also checked during pilot testing by applying a 0.5 mm thin rubberlike material (Scotch^TM^ VHB^TM^ 4905, 3M, Mapplewood, MN, USA) between the prism and the counter holder during pull-out testing. No effect on tendon draw-in and failure type could be detected. An overview of this pull-out set up is shown in Figure 2. The force was applied by a servo hydraulic cylinder and the tendon draw-in at the load free end was measured by a LVDT of type W2ATK HBM (Hottinger Baldewin GmbH (HBM), Darmstadt, Germany).

To study the bond failure it was of further interest to gain additional knowledge about the tendon draw-out at the loaded side of the tendon. Hence, two additional high-precision analogue sensors of type Baumer IPRM12I9505/S14 were mounted on top of the counter holder to measure the tendon’s draw-out. All samples tested were loaded continuously at a constant velocity of 2 mm/min until failure.

### 2.4. SEM, Microscopy and Visual Analysis

For visualization of the details on the bond failure area, electron micrographs were acquired from the surface of the CFRP tendon as well as of the corresponding concrete area failed in pull-out. This was done using a SEM of type FEI ESEM XL30 (Philips, Amsterdam, The Netherlands) at 2 kV and different magnifications.

In addition, the pull-out samples were split after loading and the failure area at the concrete’s side was examined visually. These results were recorded by a Canon PowerShot G10 camera.

The interface thickness between sand coating and CFRP tendon was determined by a Zeiss Axioskop light microscope (Carl Zeiss AG, Jena, Germany). For the microscopic analysis, a cross section of the CFRP tendons was placed in embedment resin, polished and the adhesive thickness was measured afterwards at five points around the tendon’s circumference. The measured value of 44 μm (Stdv = ±19 μm) is used as input parameter in the performed FEA. Due to the large uncertainty of this thickness, the influence of a deviating adhesive thickness on the pull-out behaviour was analysed and the result is shown in the figure of Section 5.

### 2.5. X-ray CT

X-ray computed tomography (CT) was performed at Empa’s Center for X-ray Analytics with an in-house developed X-ray micro-CT instrument based on a Viscom AG XT9225 TEP^®^ X-ray source (Viscom AG, Hanover, Germany), with a transmission target consisting of a 9 μm-thick tungsten film, and a Perkin Elmer XRD 1621 CN3 ES^®^ X-ray detector (Waltham, MA, USA), consisting of 2048 × 2048 pixels with physical size p=200 μm. Each pixel consists of CsI(Tl) scintillator layer deposited on top of amorphous silicon. The scintillator layer converts the received X-ray photons into visible light photons, which are then converted into electrical charge by the amorphous silicon material.

The X-ray source was operated at 200 kV accelerating voltage and 45 μA current, for the electron beam irradiating the target, for a total power of 9 W delivered on the target.

One attenuation-contrast X-ray CT measurement consists in acquiring several radiographs of the specimen for different orientations $\theta_{k}$ over 360°, producing a set of projection images. Tomographic reconstruction algorithms are then used to retrieve the object function $\mu \left(x^{\prime },y^{\prime },z^{\prime }\right)$, representing a 3D image of the interior of the specimen, from the set of linear projections [33].

In our case, we acquired 1441 radiographs over 360° of overall rotation of the specimen with a step of Δθ=0.25° per successive radiograph. At each orientation angle, each radiograph was actually the result of averaging over 4 of them at the same orientation, in order to increase the radiograph signal-to-noise ratio. The tomographic reconstruction was then performed with an implementation of the Feldkamp–Davis–Kress cone beam filtered back-projection algorithm [34], a type of tomographic reconstruction algorithm, available in the Octopus Reconstruction^®^ software (Ghent, Belgium) suite by Inside Matters (http://insidematters.eu/).

The 3D image (also called tomogram) consisted in a stack of 2D digital cross-sections of the volume of $\mu \left(x^{\prime },y^{\prime },z^{\prime }\right)$, orthogonal to the vertical *y*-axis and saved as 16 bit unsigned integer TIFF images.

The effective voxel size of the tomogram was $\tilde{p}=30.53\;\mu$m, corresponding to an effective spatial resolution of the images of about twice $\tilde{p}\;(61\;\mu$m).

### 2.6. Statistics

To compare the maximum bond strength means, gained during this work for different material combinations (see Scheme 1), an Analysis of Variance (ANOVA) was conducted. With the Tuckey test, significant differences between specific means were identified. The significance level α was chosen to be 0.05. If the result of Tuckey test, a *p*-value, is smaller than 0.05, the null hypothesis of no significant difference is rejected. The normal distribution of each test series was tested preceding the ANOVA.

## 3. Experimental Results and Discussion

### 3.1. Influence of Sand Coating on Bond Strength (I)

The maximum bond strength obtained with the different sand coatings (Scheme 1) was evaluated by pull out tests. The tendon material (UTS E_11_ = 136.8 GPa) as well as the concrete mixture C_ind_ was not changed during this test series.

All coating options showed a similar behaviour during the first stage of loading. A continuous increase in bond stress was observed, until the maximum bond strength was reached. The non-coated samples as well as the ones coated with fine-sand softened slightly after reaching their maximum bond strength. Afterwards, they were further pulled out at a bond stress level close to their ultimate bond strength. For all coarse-coated tendons, the recorded load dropped suddenly after reaching its maximum value and stabilized a split second later on a much lower level. However, this level was still as high as (UTS old-coarse, UHM new-coarse) or higher than (UTS new coarse) the level of the UTS fine-sand tendon during further pull out. All tendons failed by slippage and splitting of the concrete [35] prism never occurred.

**Figure 3:**
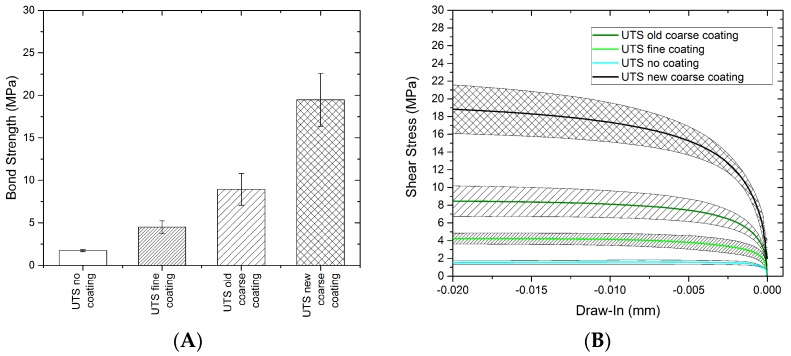
Influence of different sand-epoxy coating options on bond: (**A**) absolute bond strength comparison for different sand coating options; and (**B**) tendon draw-in behaviour. The sudden drop in load after failure is cut-off from the graphs to allow a better visualization up until failure. This graph has to be studied from right to left along its *x*-axis and shows the means and the standard deviation for all tested configurations.

The results show a clear dependency of the maximum bond strength upon the chosen sand coating. No-coating (σ_u_ = 1.73 MPa, Stdv = ±0.12) and fine-sand (σ_u_ = 4.50 MPa, Stdv = ±0.75) showed the weakest bond performance. A significant increase was reached by the old-coarse-sand (σ_u_ = 8.95 MPa, Stdv = ±1.87) and finally the new-coarse-sand (19.47 MPa, Stdv = ±3.11). All bond strength means are significantly different despite the two samples no-coating vs. fine-sand and are visualized in Figure 3A.

The particle size (fine or coarse) seems to control the bond failure mechanism (softening vs. sudden failure). If sufficiently coarse sand is applied by the aid of an epoxy resin, the bond between tendon and sand-coating is found to be fundamental for the overall bond performance. The tendon draw-in vs. bond stress, the so-called τ-δ curve, is shown for all four coating options in Figure 3B. According to these τ-δ curves, the sand coating has also an influence on the draw-in at the unloaded side of the tendon. In this case the mean draw-in at failure is found to be higher for higher maximum mean bond strength. The draw-in for the new-coarse sand reached a value of 0.04 mm while the old-coarse sand had a maximum draw-in of 0.03 mm.

**Figure 4:**
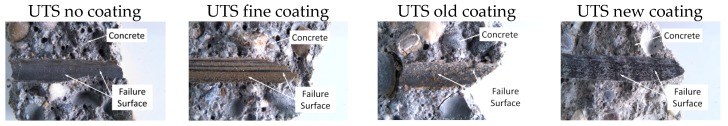
Results of visual analysis of the failure surface onside the concrete prism for no-coating, fine-coating, old-coarse-coating and new coating.

Moreover, the bond area of the four tested surface configurations was investigated visually (see Figure 4). For the two coarse coatings it was found that the sand particles stick together with their corresponding adhesive epoxy resin to the surrounding concrete. This indicates most probably that the overall bond strength is governed by the interface between the sand epoxy adhesive layer and the CFRP tendon’s surface. In the case of the fine-sand coating, the grains were also found to be present onside the concrete prism after the pull-out. However, the fine particles were not anymore completely embedded in their adhesive layer. Thus, the bond strength was dependent on the bond between the fine-sand and the coating epoxy. In conclusion, the new-coarse-sand was identified as the best option and was also applied to the specimens studied in the next sections.

### 3.2. Influence of Tendon Stiffness on Bond (II)

The aim of this program was to compare the maximum bond strength of novel UHM-CFRP sand-coated tendons (K63A12 E_11_ = 463.6 GPa, K13916 E_11_ = 509.1 GPa) to formerly-used tendons with lower stiffness [2] (UTS E_11_ = 136.8 GPa). Both UHM tendons were designed to have approximately the same fibre volume content (62%) as the reference UTS tendon. In addition, all tendons were coated with the same sand-epoxy mixture, which showed the best results in the first section of this work. The pull-out tests were performed in comparable concrete mixtures C_ind_ (UTS), C_ind_ (UHM K63A12) and in C1 (UHM K13916). All samples were loaded continuously at a constant velocity of 2 mm/min until failure.

**Figure 5:**
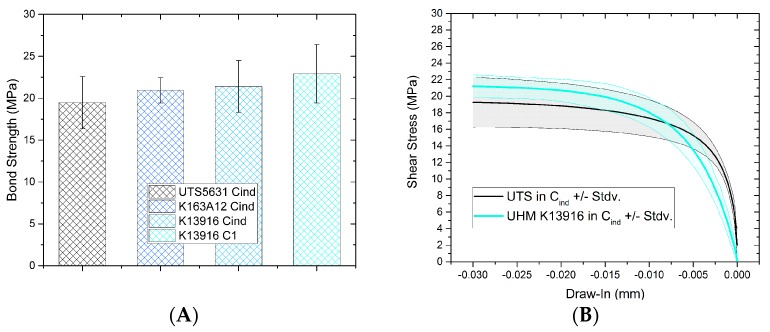
Influence of tendon stiffness on bond (UTS5631 136 GPa, K63A12 463 GPa, K13916 509 GPa). (**A**) Absolute bond strength comparison. All samples are coated with the new coarse coating and tested in C1 and C_ind_, respectively. (**B**) Tendon draw-in behaviour. The sudden drop in load after failure is cut-off from the graphs to allow a better visualization up until failure.

**Figure 6:**
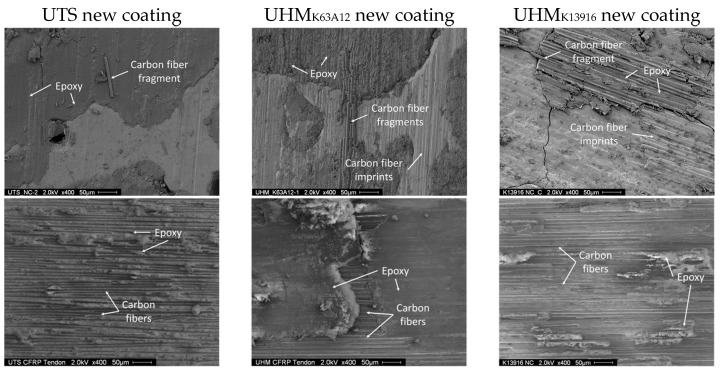
Analysis of failure area for different tendon materials. **Upper row**: Visual results indicate the sand-epoxy layer shear off onside the concrete prism; and **Lower row**: SEM images show pull-out failure surface of different CFRP tendons. This supports the hypothesis that the weak interface lies in between tendon and epoxy-sand coating, independent of the different CFRP materials used.

Similar behaviour during loading was observed for all tendons. A continuous load increase until the maximum bond strength was followed by a sudden tendon slip-out failure. The maximum bond strength of all tendons (σ_u_K63A12_ = 21.08 MPa, σ_u_K13916_ = 22.92 MPa, σ_u_UTS_ = 19.47 MPa,) was not significantly different (see Figure 5). Thus, σ_u_ seems to be mainly influenced by the adhesion of the sand-coating epoxy to the tendon’s surface. However, according to the measured τ-δ curves, the tendon draw-in seems to be influenced by the tendon’s stiffness (Figure 5B). Up to 60% of the maximum bond stress, the averaged tendon draw-in of the means of the two UHM configurations is significantly softer compared to the mean of the UTS samples. To gain more information about this characteristic, the tendon’s failure surfaces were investigated with SEM (Figure 6, lower row). For the UHM CFRP, the SEM analysis showed that the carbon fibres on the failure interface were in good order and epoxy resin was partially spalled away. Thus, the failure of the UHM-tendon occurred at the interface between the carbon fibres and the attached epoxy resin. The UTS-tendon shows a slightly different picture. Here, the fibres are still nicely in touch with the surrounding epoxy resin used as tendon’s matrix. This difference between the two tendons can be explained by different fibre surface treatments related to their production process. As before, the failed bond area inside the concrete prism was investigated visually but also by SEM. For all tendons tested, the sand coating remained visually attached as a whole (sand and epoxy resin) to the concrete. SEM reveals epoxy patches but barely any carbon fibres onside the concrete prism’s failure surface. In fact, carbon fibre fragments were found rarely and only very isolated (Figure 6, upper row). These results indicate a bond failure between sand-epoxy coating and the CFRP tendon. Again, splitting of concrete never occurred in this test series.

### 3.3. Bond Performance and Bond Failure of Sand Coated UHM CFRP Tendons (III)

The aim of this program was to compare the bond performance as well as the pull-out behaviour of novel UHM-CFRP sand-coated tendons (K13916 E_11_ = 509.1 GPa) in HPC. All tendons were coated with the same sand-epoxy mixture (Huntsman 5052 and new-coarse-sand), which previously showed the best results in the first section of this work. The pull-out tests were performed in the concrete mixture C1; after seven days (7 d) as well 28 days (28 d) after casting. The samples were loaded continuously at a constant velocity of 2 mm/min until failure.

In addition to the previous pull-out setup, the draw-out at the loaded side of the specimen was recorded by two additional sensors. This additional information should give knowledge about the whole pull-out behaviour of UHM tendons and support the analysis of the failure initiation.

**Figure 7:**
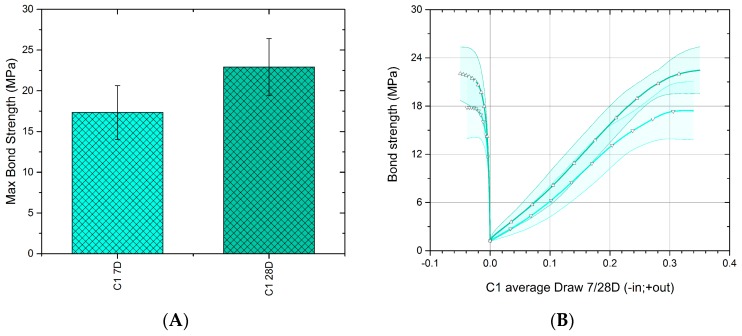
Bond performance of UHM-CFRP in C1 referenceL (**A**) maximum bond strength after seven days (7 d) and 28 days (28 d); and (**B**) tendon draw-in and -out. These graphs show the average including the standard deviation up to failure. The darker colour indicates the mixture tested after 28 d and the lighter colour the same mixture tested after 7 d. The sudden drop in load after failure is cut-off from the graphs to allow a better visualization up until failure. Starting from the point of zero draw-in-out, negative values indicate the tendon draw-in at the unloaded side and positive values the draw-out at the loaded side of the tendon.

During loading, the monitoring of the tendon draw-in and -out showed a nearly constant increase up to approximately 75% of the final loading. Afterwards, the slip increase accelerated progressively until failure. After that, the tendons were further pulled-out until the remaining pull-out load stabilized on a much lower load level. All 12 samples tested failed by a sudden slip-out after reaching their maximum bond strength. The failure happened in the same manner as all other samples with the same coarse-sand-epoxy coating tested previously. The maximum bond strength means, ranging between 17.33 and 22.92 MPa for 7 and 28 d, respectively, are visualized as a whole in Figure 7A. Even though the maximum bond strength values look different at first glance, no significant difference neither a trend could be detected for the different dates of testing by statistics. Thus, the maximum bond-strength averaged over all samples of this test series is 20.13 MPa. The visualization of the corresponding τ-δ curves in Figure 7B is based on sensor data. Again, in these figures, the mean values of the tested samples and their standard deviation are visualized.

The τ-δ curves suggest that the tendon draw-out rate of samples tested after 7 days is higher compared to the draw-out rate after 28 days. However, based on the shown standard deviations, the effect of specimen age is statistically not significant. Furthermore, the slippage and the tendon draw-in rate at the unloaded side of all samples seems not be influenced by the concrete’s age at loading.

**Figure 8:**
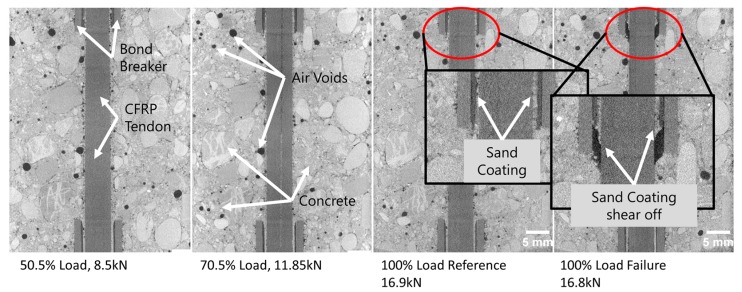
2D vertical, digital vertical cross-sections from the X-ray tomograms acquired after loading the sample up to 50.5%, 70.5%, Reference 100% and 100% failure, from left to right, respectively.

To gain more information of the bond failure initiation and to detect possible internal cracks at low loads, one sample was investigated at different load levels by X-ray CT. This sample was loaded in the same manner as all other tested samples to the following load levels: 50.5%, 70.5%, 100% no failure and 100% including failure. After reaching each level, the load was released instantly and the sample was transferred from the pull-out test setup into the X-ray CT setup. This procedure was repeated after each loading and the generated X-ray scans (Figure 8) were analysed visually. Up to 100% of loading, neither slippage nor cracks could be detected with the given setup configuration. Only after loading the sample until its sudden slip-out failure, the tendon slip could be visualized and was clearly indicated in the interface between the sand-epoxy layer and the CFRP tendon (see right side of Figure 8). The loading until 100% was possible due to online monitoring of the tendon draw-in. As visualized in Figure 7B, the tendon draw-in rate rapidly increases before failure. At this point, the loading was stopped and immediately released manually.

## 4. Numerical Modelling

The pull-out behaviour found in the experimental sections of this work is collated and modelled by the Finite Element Method in the commercial software Abaqus 6.14. This model should show the applicability of the idea to reduce the failure behaviour down to the adhesive properties between CFRP tendon and sand-coating epoxy resin. Furthermore, this numerical model allows additional investigations on possible bond-influencing parameters and clarifies the origin of the experimentally-observed softening of the draw-in behaviour of UHM-tendons when compared to the behaviour of UTS tendons.

### 4.1. Finite Element Analysis

**Figure 9:**
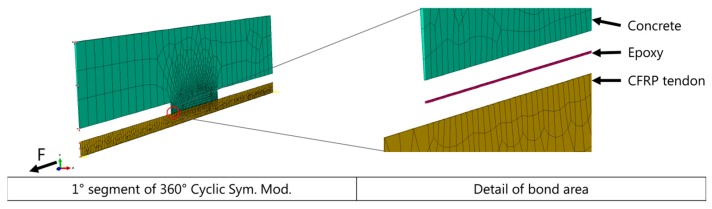
Overview of all components in the FEA.

The structure of the implemented model is based on the idea of cyclic symmetry. To save computational cost, only a one-degree segment of a cylindrical pull-out prism is modelled. This is a simplification as the original prisms tested have a rectangular cross section. The FEM results show that the shear stress, transmitted from the tendon to the concrete, decreases very fast in radial direction. Hence, the modelled pull-out behaviour is expected to be independent of the actual cross section of the concrete prism. The model consists of three different areas which are related to the main materials and assembled as shown in Figure 9. As found in the experimental work, the sand coating was never ripped out of the concrete prism. Thus, these particles are not modelled in detail and are consequently neglected. However, the bond between the sand coating and the CFRP tendon is modelled by a very thin layer of epoxy resin. The thickness of this layer was chosen according to light microscopy measurements and an explanation of this method can be found in the materials and methods section of this work. In this FEA, only the new coarse coating was considered for the comparison between UTS- and UHM-CFRP.

In this model, the bond between the epoxy layer and the concrete is assumed to be perfect. Between the tendon’s surface and the epoxy layer, a surface-to-surface contact interaction of type surface-based cohesive behaviour is embedded. The main parameters controlling this cohesive behaviour are the cohesive stiffness and the damage initiation. Due to the explicitly modelled epoxy layer, which takes the compliance into account, and the infinitely thin interface between tendon and epoxy, the cohesive stiffness should be set to an infinitely high value. In the presented FEA, the cohesive stiffness was defined to 2 × 10^6^ N/m^3^. This value was chosen to achieve a good numerical convergence and to avoid possible issues usually related to high cohesive stiffness values [36]. A sensitivity analysis related to the cohesive stiffness value was performed and can be found in the summary and discussion section. For the damage initiation, the experimentally-found bond strength of 22.9 MPa was implemented.

Overall, the model is constrained by a boundary condition of type Encastre, which constrains all displacements and rotations of the corresponding nodes, on the left side of the concrete prism to represent the counter holder. The load is applied, equally distributed on the front face of the CFRP tendon looking to the left in Figure 9, and directed along the longitudinal axes of the prism. In addition, the movement of the CFRP tendons front face is blocked in transverse directions. The concrete and the CFRP tendon are modelled as three dimensional linear brick elements with reduced integration of type C3D8R. The epoxy layer is modelled as fully integrated three dimensional linear brick elements of type C3D8. These linear brick elements are in general well suited for contact problems and thus were chosen here for good numerical convergence. To reach a good accuracy of the cohesive behaviour along the bond length, the mesh is refined locally. The elements along the bond interface are 0.5 mm long. All parts are assembled without any overclosure and shrinkage of the concrete during hardening is neglected.

For this task, the concrete material was assumed to be homogeneous and isotropic material with a linear elastic behaviour (Young’s modulus E = 40.59 GPa, see Table 1; Poisson ratio ν = 0.178, following [1]). The epoxy layer (Huntsman 5052), was modelled to be isotropic linear elastic (E = 2.63 GPa, ν = 0.35) up to 30 MPa of stress. Afterwards, a nonlinear behaviour was implemented up to a failure stress of 70 MPa with the corresponding failure strain of 4.56%. The linear elastic as well as the nonlinear behaviour was gained by tensile testing of the related Hunstamn 5052 epoxy resin according to EN ISO 527-2:2012 (Determination of tensile properties).

### 4.2. FEM Validation

**Figure 10:**
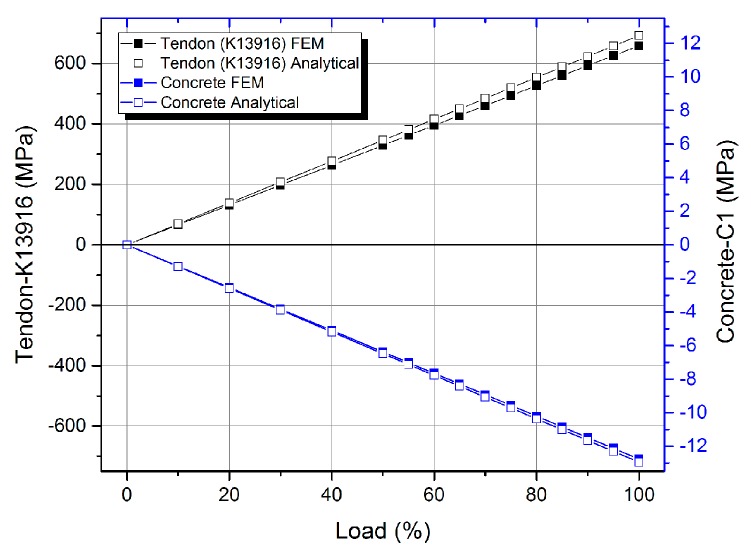
Validation of FE model. The calculated stresses inside the concrete as well as the CFRP tendon are compared. The blue graph indicates the concrete and the black graph represents the values of the CFRP tendon.

The validity of the implemented finite element (FE) model was checked in a simple comparison. The stresses, calculated values based on experiments inside the CFRP-tendon and the concrete-prism, were compared to their counterpart in the FEA. In both cases, a good agreement of model and experiment is present (Figure 10). However, it is important to notice that this simplified verification cannot cover and thus verify the influence of all input parameters. The sensitivity of the simulation to two main input parameters such as the adhesive thickness and the cohesive stiffness are discussed in the summary section.

### 4.3. Results

**Figure 11:**
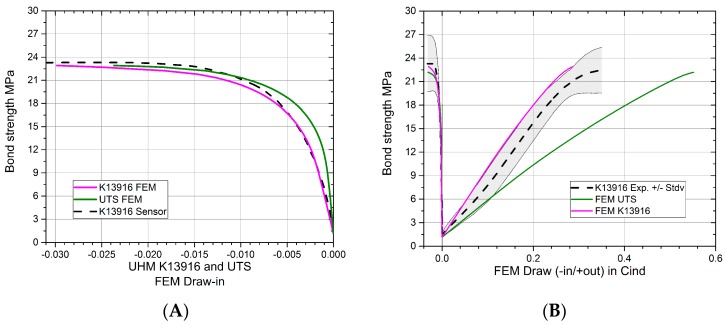
Tendon draw FEM compared to related Sensor measurements. (**A**) Draw in for FEM UTS and FEM UHM_K13916_. Results show the same trend as experiments in terms of the higher tendon draw in for the UHM tendon. Sensor data is shown as reference. (**B**) Draw-in and -out modelled using C_ind_, UTS and UHM_K13916_. Sensor 28 d of UHM_K13916_ properties are shown as a reference.

Overall, the FEA showed good agreement with the experimental results. As seen before in the experiments, the tendon draw-in/-out starts nearly linear at low pull-out loads but increases rapidly thereafter, see Figure 11A. As expected, the simulated draw-rate at the loaded end of the tendon is much higher compared to the unloaded end. The only noticeable difference between FEA and experiments was found in the early stage of the tendon draw-out at the loaded side of the tendon. Here, the experiments showed a slightly weaker s-shaped draw-out in the early stage up until 9 MPa of bond strength. Afterwards and logically at a certain off-set, the slope and the shape of both curves are again comparable. This effect is most likely due to settlement or orientation effects during loading, e.g., minimal local concrete crushing at the contact to the steel counter holder, loosening effects of small cement paste leftovers which were previously in contact with the tendon or slight tilting between the draw-out sensors and the counter holder.

Furthermore, the FEA was used to investigate the origin of the experimentally-found differences in the tendon draw-in for UHM and UTS tendons. For this purpose, only the CFRP material was changed, from K13916 (UHM) to a UTS CFRP, in the simulation. Without adapting any other parameters, such as epoxy material, concrete type or adhesive layer thickness, the FEA result already indicated the same behaviour as found experimentally. This is visualized in Figure 11B, in which the UHM CFRP tendon showed a higher tendon draw-in rate at low loads if compared to the UTS CFRP tendon. In addition, the experimentally-gained draw-in curve of the K13916 UHM CFRP tendon is plotted in the same graph as a reference.

**Figure 12:**
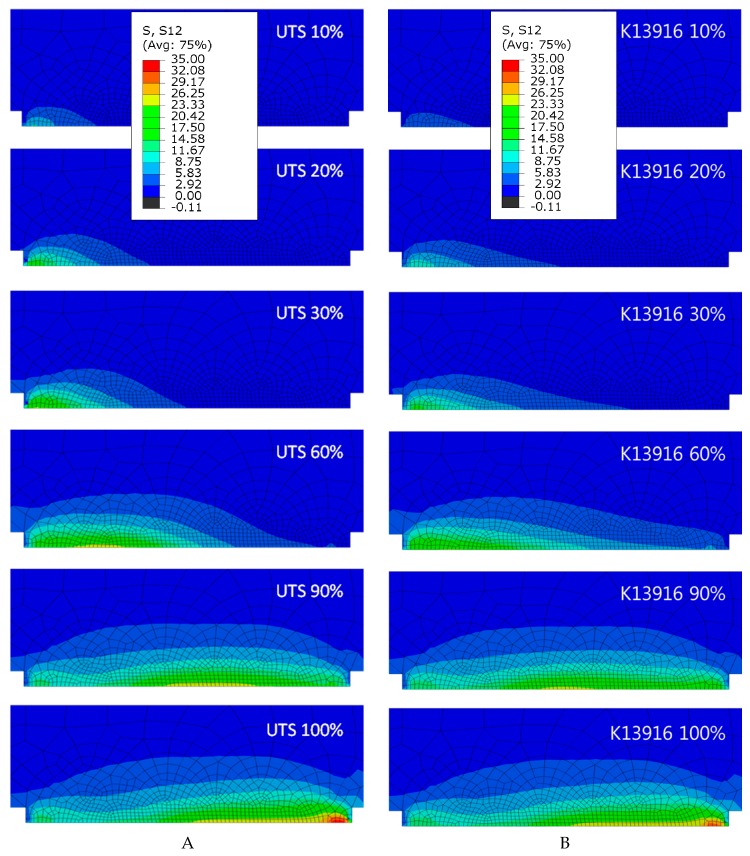
Shear stress distribution in longitudinal direction of the prism up to 22.39 MPa (100%) bond stress: (**A**) modelled using C_ind_ and a UTS tendon in pull-out; and (**B**) modelled using C_ind_ and UHM_K13916_ tendon in pull-out.

Based on this finding, it was suggested that the stress transfer in the bond area during a pull-out test should be more widespread at low loads for the UHM CFRP tendon in comparison to the UTS CFRP tendon. The visualization of the shear stresses inside the concrete prism, at different load levels and along the bond area confirmed this assumption (see Figure 12). Moreover, the transferred peak shear stress in the concrete is higher for the application of UTS CFRP tendons.

**Figure 13:**
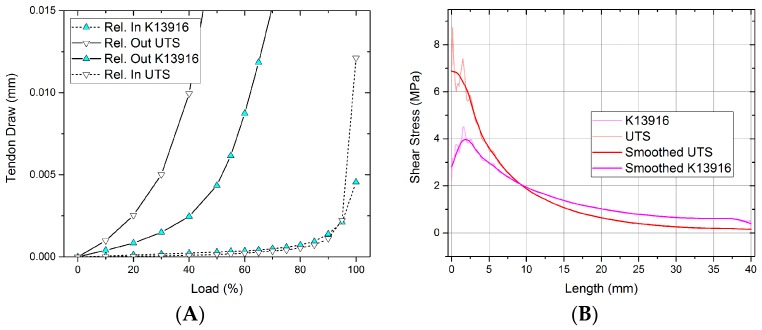
Influences on tendon draw-in and -out behaviour: (**A**) relative displacement between concrete and CFRP at the beginning (draw-out) and the end (draw-in) of the bond length; and (**B**) shear stress in the middle of the sand-epoxy segment, *t* = 44 μm, along the bond length for two CFRP materials at 10% load.

A deeper look into the modelled adhesive layer pointed into the same direction. Figure 13A shows the relative displacement between the corresponding nodes at the concrete prism and the CFRP tendon, once at the loaded and once at the unloaded side. Here, it was found that the displacement for the UTS tendon was much larger and consequently, in the undamaged stage, the dependent shear stresses of the adhesive layer as well. Figure 13B illustrates this fact, in which the shear stress in the middle of the adhesive layer is plotted along with the bond length for both tendon materials. The found results are in analogy to the shear stress analysis in adhesive layers as summarized by, e.g., da Silva et al. [37]. In [38], Tsai et al. showed for the specific case of a double lap-shear-test that the shear stress in the adhesive layer is significantly dependent on the stiffness of the adhered parts. The same dependency was found in the presented FEA. Note, the found shear stress distribution does not necessarily correlate to a failure criterion as the failure initiation is most probably a local phenomenon. A subsequently performed sensitivity analysis, by separately changing every input material property of the CFRP tendon from UHM to UTS, revealed that only the change of the longitudinal stiffness from 137 to 509 GPa showed a significant influence on the tendon draw behaviour.

## 5. Summary and Discussion

The size of sand particles in the coating of the CFRP tendon has a significant influence on the bond failure mechanism. The tendons with either no coating or fine-sand coating showed a low bond strength but also a more continuous failure behaviour. However, if sufficiently coarse sand (>0.5 mm) and sufficiently high concrete strength (>85 MPa) were used, a sudden-slip failure at a much higher load values, as found for the two coarse coatings, was observed. In this case, the maximum pull-out strength reached values of 12–22 MPa and was limited by the bond properties of the corresponding epoxy-CFRP interface. This assumes that the bond between the coarse sand particles and the epoxy adhesive is much higher than the bond at the named interface. Both failure behaviours as well as the visual analysis of the failed surfaces confirm the experimental results shown in [18].

In addition, the applied different sand-epoxy combination used for the new-coarse coating showed an additional increase in the maximum bond strength. This effect is most probably related to the better adhesion to the tendon’s surface of the Huntsman 5052 in comparison to the Bakelite epoxy resin. Furthermore, in the case of the brittle failure, the remaining load during further tendon pull out is slightly higher when compared to the load needed for the uncoated or fine-coated tendons. This effect was also found in [18], but was not further discussed. One explanation could be that this increase is related to the additional peel of the sand coating which is happening in these samples.

The tendons’ longitudinal stiffness does not affect the maximum bond strength. This was shown over a broad range of longitudinal tendon stiffness (136 to 509 GPa). Restrictions may also apply for low strength concrete as well as for a pull-out failure different than the sudden slip-out. For instance, it was shown in [17] that for concrete compressive strength values around 15 MPa the bond failure will most probably appear inside the concrete prism. However, up to 60% of the max pull-out strength the tendon draw-in was influenced by the tendon’s stiffness. Contrary to expectation, the stiffer tendon showed softer draw-in behaviour. This effect was investigated later in the FEA and found to be related to the longitudinal tendon stiffness only. This effect might also influence the transfer behaviour in a pre-stressed element and thus needs to be considered for the following work.

It was mentioned in the literature [17] that, above a certain minimum compressive strength of the concrete, the concrete strength does not influence the pull-out behaviour of CFRP tendons. However, these studies investigated mainly ordinary concrete mixtures and tendons of a much lower stiffness. Here, it was shown that this assumption is also valid for sand-coated UHM CFRP tendons in combination with a HPC.

Earlier, in [16,17], the failure interface was already directed to be either inside the outer CFRP layers or in the coating of the tendon. For the sudden-slip failure of UHM CFRP tendons, it is shown in this work, by the aid of X-ray tomography, SEM and double notch shear (DNS) testing that the failure occurred directly at the interface between the coating epoxy and the tendon.

**Figure 14:**
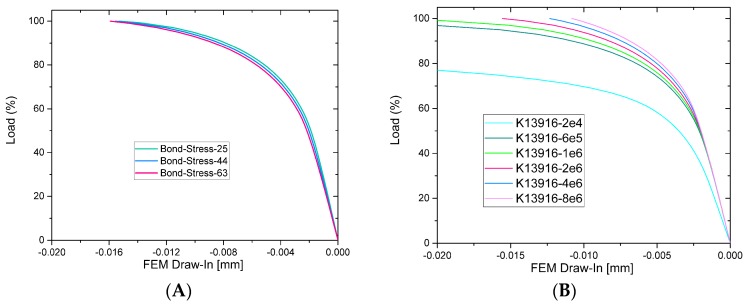
Sensitivity of FE model. (**A**) Thickness of the adhesive layer. In this visual, the adhesive thickness in μm is indicated by the number behind the label, e.g., Bond-Stress-25 represents an adhesive layer thickness of 25 μm; (**B**) Variation of cohesive stiffness and their influence on the tendon draw-in behaviour.

In general, the subsequently-performed FEA is based on this finding with the chosen position of the cohesive surface interaction between the ideal CFRP tendon and the adhesive layer. This implies that the FEA does not give any information about the local failure mechanisms. The FEA assumes that the local failure initiations, as most probably found in the experiments, act together in a global manner. Furthermore, the FEA showed also a certain dependency from important input parameters such as the adhesive thickness and the cohesive stiffness, see Figure 14A,B respectively. In these graphs it was clearly shown that both values could possibly influence the draw-in behaviour significantly. With the performed 2D-Microscopy analysis of the sand coated tendon’s cross-section, it was not possible to gain more precise information. For a complete description of the cohesive contact layer properties, a combination of 3D experimental and a finite element analysis would be necessary.

## 6. Conclusions

Based on the test results of this study and the finite element analysis performed, the following conclusions can be drawn:

- The type of sand coating has a significant influence on bond for the used CFRP tendons. It is recommended to use a coarse sand (0.5–1.5 mm) to ensure the sudden-slip failure and thus the highest possible bond strength. Although this failure is brittle, it can be favoured over the soft behaviour due to the much higher level of absolute bond strength and the fact that a concrete element should not at all fail due to tendon pull-out.
- The highest bond strength in the given configuration seems to be dependent on the strength of the adhesive interface. The combination of X-ray, SEM, and visual analysis of the bond failure surfaces allowed locating the bond failure between CFRP tendon and the sand epoxy layer.
- The stiffness of a unidirectional CFRP tendon does not affect the maximum bond strength but it does influence the draw-rate in the corresponding τ-δ curve. In this case, the draw-in rate and the shear stress transfer behaviour between tendon and concrete are mainly controlled by the stiffness in pull-out direction of the related materials. Thereby, the most crucial condition of a good bond, according to [11], for the design of load introduction areas is fulfilled for UHM CFRP tendons.

It can be summarized that the results of this work motivate a further investigation of the proposed beneficial material combination (UHM-CFRP and HPC). The focus of the following research will be the investigation of the prestress transfer behaviour. In addition, it will also be very interesting to see how these materials perform under long term and how creep effects will affect the performance.

## Figures and Tables

**Table 1:** Properties of Concretes.

CONCRETE	Compressive strength (Mpa)	Tensile strength (MPa)	Tensile young’s modulus (GPa)	Density (kg/m^3^)
Standard	EN12390-3:2009	SIA 2052 (adapted)	SIA 2052 (adapted)	EN12390-3:2009
#of samples	3 (15 C_ind_)	5	5	3 (15 C_ind_)
C_ind_	89.18 ± 2.52	6.58 ± 0.40	40.59 ± 2.11	2413 ± 132.18
C1	85.70 ± 1.14	6.59 ± 0.84	42.26 ± 5.48	2299 ± 2.08

**Table 2:** Properties CFRP Tendons.

CFRP	Shear strength (MPa)	σ_τmax_ (MPa)	E11 (GPa)	ε_τmax_ (%)	Poisson ratio	FVC (%)
Standard	DIN 65148	EN 2561	EN 2561	EN 2561	EN 2561	EN 2564
UTS5631	30.00 ± 0.86	1576.67 ± 111.36	136.84 ± 4.59	1.152 ± 0.043	0.30	60.81
K63A12	28.69 ± 4.47	1029 ± 109.72	463.64 ± 28.16	0.230 ± 0.037	0.26	60.43
K13916	31.83 ± 2.47 ^2^	1561.98 ± 68.05 ^1^	509.12 ± 13.49 ^1^	0.307 ± 0.017	0.22	67.08

^1^ data equivalent to [29]; ^2^ three test specimens.
